# Lipid-Based Nutrient Supplements: How Can They Combat Child Malnutrition?

**DOI:** 10.1371/journal.pmed.1001314

**Published:** 2012-09-18

**Authors:** Kathryn G. Dewey, Mary Arimond

**Affiliations:** University of California, Davis, Davis, California, United States of America

## Abstract

Kathryn Dewey and Mary Arimond discuss new research in *PLOS Medicine* that assesses the effect of blanket provision of ready-to-use supplementary food to children at high risk of malnutrition in Chad, and highlight some of the challenges of investigating the efficacy of supplementary foods for malnourished children.

**Linked Research Article**

This Perspective discusses the following new study published in *PLOS Medicine*:

Huybregts L, Houngbé F, Salpéteur C, Brown R, Roberfroid D, et al. (2012) The Effect of Adding Ready-to-Use Supplementary Food to a General Food Distribution on Child Nutritional Status and Morbidity: A Cluster-Randomized Controlled Trial. PLoS Med 9(9): e1001313. doi:10.1371/journal.pmed.1001313

Lieven Huybregts and colleagues investigate how supplementing a general food distribution with a fortified lipid-based spread during a seasonal hunger gap in Chad affects anthropometric and morbidity outcomes for children aged 6 to 36 months.

Interest in the use of lipid-based foods to combat child malnutrition has grown, accelerated by the success of ready-to-use therapeutic foods (RUTF) for the treatment of severely malnourished children [1]–[3]. This has motivated the development of a range of lipid-based products designed for different purposes, including treatment of moderate acute malnutrition (MAM) (wasting, or weight-for-height below -2 SD of the standard) and prevention of wasting or stunting (low height-for-age). With more lipid-based products comes a diversity of terminology, challenges in comparisons across products, and confusion about the appropriate use of the term RUTF.

Lipid-based products vary along a number of dimensions but most notably in energy dose and concentration of micronutrients. RUTFs are designed to achieve rapid nutritional recovery and are thus provided in large amounts (200–300 g/d), temporarily replacing most or all other foods aside from breast milk. By contrast, lipid-based nutrient supplements (LNS) designed for prevention of wasting or stunting are meant to be given in much smaller amounts (20–50 g/d), which means that they are more concentrated in micronutrients and cost less. At the low end of this range, “small-quantity” LNS such as Nutributter, which provide a daily ration of only 20 g (4 teaspoons), are considered suitable for “home-fortification” of local foods (with micronutrients, essential fatty acids and a small amount of protein) and leave more “room” for other foods in the diet. The term “ready-to-use supplementary food” (RUSF) has been inconsistently used, sometimes referring only to products used for treatment of MAM, and sometimes also to refer to products used for prevention of MAM. Efforts to standardize use of terminology are underway.

Unlike use of RUTF, which is currently recommended for treatment of severe acute malnutrition [4], other lipid-based products for different purposes are still under study—comparative effectiveness and cost-effectiveness are not yet established. In the meantime, and especially in emergencies, international agencies and other humanitarian actors must make choices in the absence of robust evidence. These decisions are based on an analysis of the situation as well as the programming and product options that are available and guidance is beginning to emerge [5],[6].

Vexing the field is ongoing concern and controversy about the role of lipid-based products in the diet of infants and children, their potential to displace breast milk or diverse local diets, whether and how imported or processed foods should be used to prevent or treat malnutrition, inappropriate commercial promotion of infant foods, and finally the cost of the products, which raises concerns both about sustainability and trade-offs/opportunity costs. These issues are complex, and some are more or less salient depending on the daily ration size of the product, and on whether products are distributed through programmatic channels, through private sector marketing, or some mix of the two. Research to date provides some evidence that compared to local porridge, use of up to 50 g/d of LNS does not displace breast milk [7],[8]. We encourage continued attention to these critical issues. New research in this week's *PLOS Medicine* [9] adds to the evidence base and our understanding of the role of lipid-based products in child malnutrition.

## A New Study In Chad

One approach that has been studied by several research groups is the blanket provision of “medium-quantity” LNS (e.g., ∼50 g/d) to children at high risk of malnutrition. In this issue of *PLOS Medicine*, Lieven Huybregts and colleagues describe the results of a cluster-randomized pragmatic trial conducted in Chad to evaluate this strategy in the context of a food distribution program [9]. In this case, the product (Plumpy'Doz, 46 g/d) was added to a package of monthly household food rations for a period of 4 months and targeted to children 6–36 months of age (*n* = 1038). This intervention did not significantly reduce the incidence of the primary outcome, wasting, which is perhaps not surprising given that the control group also received the household ration, designed to cover 86% of daily energy requirements. However, the intervention group exhibited greater growth in height, lower prevalence of anemia, and less morbidity (29% reduction in incidence of diarrhea, 23% reduction in incidence of fever) than the control group. Because the *quantity* of food (energy provided) was presumably not limiting in either group, this speaks to the importance of dietary *quality* for infants and young children.

Three other trials have investigated the blanket provision of a similar quantity of LNS to that used in Chad [10]–[12]. In all three studies, two from Malawi and one from the Democratic Republic of Congo (DRC), infants were enrolled at 6 months of age and were randomized to the intervention group(s) that received LNS or to a control group that received another type of food supplement intended to provide an equivalent amount of energy. These studies have produced mixed results for weight and height gain, and it seems likely that some of this variation may be explained by differences across the trials in the foods provided to both the control and intervention groups. For example, control groups received food that was fortified with micronutrients (both Malawi studies) [10],[12] or enriched with fish powder (DRC) [11], which contrasts with the situation in Chad where the control group received unfortified staple foods. Furthermore, in the first Malawi study [10] (as in Chad), the LNS included dried skim milk powder as a key ingredient, whereas in the second Malawi study [11] the LNS did not include any milk and in the DRC [12] the amount of milk was about one-fifth of the amount in the LNS used in the other two studies. Other research has suggested that milk products may increase child growth by stimulating growth factors such as IGF-I [13],[14]. The phytate content of the products also differed, which can affect the absorption of key nutrients such as iron and zinc.

## Where Next For Child Nutritional Supplements?

These studies illustrate that conclusions regarding the efficacy of various types of LNS may depend on the target group (age range), baseline prevalence and type of undernutrition, study design (e.g., type of control group; duration of intervention), and ration size and composition of the products being evaluated. Large variability across these dimensions in the small number of studies conducted to date makes it impossible to generalize.

There is clearly a need for additional research to understand the potential growth-promoting effect of certain ingredients in LNS (e.g., milk powder, essential fatty acids). The new study by Huybregts et al. [9] is an important contribution to the evidence base. Meanwhile, programmatic experience with the blanket provision of both “medium-quantity” and “small-quantity” LNS is growing, and in some cases is providing further evidence regarding effectiveness for prevention of wasting and stunting [15]. While that evidence is not as robust as the results of randomized controlled trials, careful attention to study design and data analysis should yield interpretable findings that are immediately relevant to real-world settings. Programmatic studies are also essential for understanding optimal ration size for the target age group and how best to integrate the appropriate use of high-quality fortified products with the other essential components for improving infant and young child nutrition, such as promotion of continued breastfeeding, dietary diversity, responsive feeding practices, and attention to hygiene and sanitation [5],[6]. Finally, high-quality programmatic studies can help provide urgently needed information on the cost and comparative cost-effectiveness of different integrated strategies for filling nutrient gaps and promoting healthy growth.

## Abbreviations

DRC: Democratic Republic of Congo
LNS: lipid-based nutrient supplements
MAM: moderate acute malnutrition
RUSF: ready-to-use supplementary food
RUTF: ready-to-use therapeutic foods
